# Cerebrospinal fluid markers of neuroinflammation in delirium: A role for interleukin-1β in delirium after hip fracture

**DOI:** 10.1016/j.jpsychores.2014.06.014

**Published:** 2014-09

**Authors:** Eleanor Cape, Roanna J Hall, Barbara C van Munster, Annick de Vries, Sarah EM Howie, Andrew Pearson, Scott D Middleton, Fiona Gillies, Ian R Armstrong, Tim O White, Colm Cunningham, Sophia E de Rooij, Alasdair MJ MacLullich

**Affiliations:** a Edinburgh Delirium Research Group, University of Edinburgh, Edinburgh, Scotland, UK; b Centre for Cognitive Ageing and Cognitive Epidemiology, University of Edinburgh, Edinburgh, Scotland, UK; c Department of Geriatrics, Western General Hospital, Edinburgh, Scotland, UK; d Department of Medicine, Amsterdam Delirium Study Group, Academic Medical Centre, University of Amsterdam, Amsterdam, The Netherlands; e Department of Geriatrics, Gelre Hospitals, Apeldoorn, The Netherlands; f University of Leuven, Leuven, Belgium; g Centre for Inflammation Research, University of Edinburgh, Edinburgh, Scotland, UK; h Department of Trauma and Orthopaedics, Royal Infirmary of Edinburgh, Edinburgh, Scotland, UK; i Department of Anaesthetics, Royal Infirmary of Edinburgh, Edinburgh, Scotland, UK; j School of Biochemistry and Immunology, Trinity College Dublin, Dublin, Ireland

**Keywords:** Delirium, Cerebrospinal fluid, Inflammation, Interleukin-1β, Interleukin-1 receptor antagonist

## Abstract

**Objective:**

Exaggerated central nervous system (CNS) inflammatory responses to peripheral stressors may be implicated in delirium. This study hypothesised that the IL-1β family is involved in delirium, predicting increased levels of interleukin-1β (IL-1β) and decreased IL-1 receptor antagonist (IL-1ra) in the cerebrospinal fluid (CSF) of elderly patients with acute hip fracture. We also hypothesised that Glial Fibrillary Acidic Protein (GFAP) and interferon-γ (IFN-γ) would be increased, and insulin-like growth factor 1 (IGF-1) would be decreased.

**Methods:**

Participants with acute hip fracture aged > 60 (N = 43) were assessed for delirium before and 3–4 days after surgery. CSF samples were taken at induction of spinal anaesthesia. Enzyme-linked immunosorbent assays (ELISA) were used for protein concentrations.

**Results:**

Prevalent delirium was diagnosed in eight patients and incident delirium in 17 patients. CSF IL-1β was higher in patients with incident delirium compared to never delirium (incident delirium 1.74 pg/ml (1.02–1.74) vs. prevalent 0.84 pg/ml (0.49–1.57) vs. never 0.66 pg/ml (0–1.02), Kruskal–Wallis p = 0.03). CSF:serum IL-1β ratios were higher in delirious than non-delirious patients. CSF IL-1ra was higher in prevalent delirium compared to incident delirium (prevalent delirium 70.75 pg/ml (65.63–73.01) vs. incident 31.06 pg/ml (28.12–35.15) vs. never 33.98 pg/ml (28.71–43.28), Kruskal–Wallis p = 0.04). GFAP was not increased in delirium. IFN-γ and IGF-1 were below the detection limit in CSF.

**Conclusion:**

This study provides novel evidence of CNS inflammation involving the IL-1β family in delirium and suggests a rise in CSF IL-1β early in delirium pathogenesis. Future larger CSF studies should examine the role of CNS inflammation in delirium and its sequelae.

## Highlights

• Exaggerated central inflammatory responses to stressors are implicated in delirium.
• We examine cerebrospinal fluid (CSF) and serum cytokines in hip fracture patients.
• We find increased CSF IL-1β in those about to develop delirium.
• We find increased CSF interleukin-1 receptor antagonist in those with active delirium.

## Introduction

Delirium is a serious, common neuropsychiatric condition with major implications for morbidity and mortality in frail older people [1]. It is triggered by peripheral insults such as infection, trauma or surgery, the pathways linking these peripheral processes with altered central nervous system (CNS) functioning and consequent delirium are still poorly understood. Studies using animal models have established that acute systemic inflammation, induced by peripheral lipopolysaccharide (LPS) or E. Coli infection, induces deficits in hippocampal-dependent memory in rodent brains made vulnerable by neurodegenerative disease [2] or age [3]. This disruption is transient [2], and associated with greater induction of mRNA for pro-inflammatory cytokines [2,3] and higher interleukin-1β (IL-1β) cytokine levels in the hippocampus [4]. Furthermore, interleukin-1 receptor antagonist (IL-1ra) has been shown to block LPS-induced working memory deficits and systemically administered IL-1β is sufficient to induce similar deficits in an animal model of delirium during dementia [5].

Recent studies have provided direct evidence of a relationship between cytokine levels and delirium. Increased serum levels of the pro-inflammatory cytokines interleukin-6 and -8 (IL-6, IL-8) were found in elderly hip fracture patients with delirium [6,7]. Increased serum interferon gamma (IFN-γ), and decreases in the anti-inflammatory insulin-like growth factor 1 (IGF-1) and IL-1ra have been demonstrated in elderly medical patients with delirium [8,9]. In cerebrospinal fluid (CSF), significantly higher levels of IL-8 have been demonstrated in delirium after hip fracture [10]. Examination of CSF in search of the pathogenesis of delirium has potential advantages due to its proximity to the brain and its immune-privileged position behind the blood brain barrier (BBB).

The mental status changes often referred to as ‘sickness behaviour syndrome’ are thought to represent a coordinated set of behavioural changes which, in health, promote survival [11]. Peripheral and central production of cytokines leading to altered neurochemical signalling may partly underpin this syndrome, with a potentially important role for IL-1β [11]. There are multiple routes by which systemic inflammatory stimuli signal to the brain. Peripheral IL-1β can induce the synthesis of prostaglandins, which cross the BBB [12], and IL-1β itself can enter the CNS via an active transport with a saturable transport mechanism [13,14], and via the circumventricular organs which lack a patent BBB. IL-1β may also induce hyperpermeability of brain microvascular endothelium, which forms the BBB [15]. Importantly, systemically administered IL-1β has been shown in rodents to cross into the CNS, so it cannot be assumed that CSF IL-1β has come from CNS synthesis [14].

These well-conserved, protective, sickness behaviour mechanisms may prove pathological if occurring over a long time period or in the context of neurodegeneration [16]. Studies in an animal model of neurodegeneration have shown that hippocampal microglial cells (the brain's resident macrophages) are primed by primary pathology to produce more IL-1β in response to peripheral inflammatory challenge [17]. Similar immune cell changes in vulnerable brains may underlie an exaggerated inflammatory response to peripheral injury, seen in clinical practice when a seemingly minor inflammatory insult induces a significant deterioration in the elderly and those with dementia, potentially including delirium and the hastening of cognitive and functional decline [18,19].

This study aimed to investigate five biomarkers known to be involved in the neuroinflammatory process in rodents. We hypothesised that delirium would be associated with increases in the pro-inflammatory cytokines IL-1β and IFN-γ, and a marker of astrocytosis GFAP, and decreases in anti-inflammatory IGF-1 and IL-1ra, and that this would be shown in the CSF.

## Methods

### Participants

Patients over the age of 60 years awaiting surgery for hip fracture were recruited in two university teaching centres: Royal Infirmary of Edinburgh (two studies) and Amsterdam Medical Centre during November 2007 to January 2010 and October 2005 to February 2008 respectively. Patients were considered eligible unless they had recent head injury, could not communicate fluently in English or Dutch, or were moribund on admission, were receiving steroid treatment or in institutional care (second Edinburgh study), had surgery with general anaesthetic planned, or in whom informed consent could not be obtained. Informed written consent was sought from patients, or a proxy in the case of patients lacking capacity to provide consent. Ethical approval was obtained from the Scotland A Research Ethics Committee or the Academic Medical Centre Ethics Committee in Amsterdam, the Netherlands.

### Delirium assessments

Delirium assessments for each patient were carried out pre-operatively on the day of surgery, followed by a post-operative assessment 3–4 days later. Assessment consisted of mental status assessment with cognitive tests, examination of case notes and discussion with clinical staff, leading to a DSM-IV [20] diagnosis of delirium assessed with the CAM [21]. Delirium cases were defined as delirium present pre-operatively and active at the time of sample collection (prevalent) or delirium not present pre-operatively but developing post-operatively (incident). Delirium was also broadly classed as being present or absent at any stage during the peri-operative period. Casenotes were searched for a pre-existing diagnosis of dementia, and in the second Edinburgh study and Amsterdam study also, the Informant Questionnaire on Cognitive Decline in the Elderly [22] was used to ascertain premorbid cognitive status, using an average score of 3.44 to indicate pre-existing dementia [23]. The Charlson co-morbidity index was used to assess the burden of co-morbid disease [24].

### Sample acquisition, processing and storage

#### Cerebrospinal fluid (CSF)

Samples were taken before administration of spinal anaesthesia and kept at 4 °C before being spun at 1000 g for 10 min at 4 °C. Supernatants were stored at − 80 °C.

#### Blood

In a subgroup of participants in the Edinburgh study (N = 24), 10 ml blood was collected. Samples were taken at the same time as the lumbar puncture, kept at 4 °C and spun within three hours at 3000 g for 10 min at 4 °C. Aliquots of serum were stored at − 80 °C before determination.

### Assays

Concentrations of IL-1β, IL-1ra, IFN-γ and IGF-1 were measured using normal sensitivity Quantikine ELISA kits from R&D systems (Abingdon, UK), and GFAP concentration using an ELISA kit from Biovendor (Modrice, Czech Republic). Samples, both serum and CSF, were thawed directly before use and processed according to manufacturer's instructions following the serum/plasma procedure. Samples were assayed singly or in duplicate if sufficient sample was available. The IGF-1 assay involved a 100-fold dilution as a pre-treatment step, and IL-1β samples were diluted 2-fold. The IL-1β bottom standard was also diluted 2-fold in order to measure low concentration in the samples. The manufacturer's recommended lower limits of detection (LoD) were 1 pg/ml, 6.26 pg/ml, 8 pg/ml, 26 pg/ml and 45 pg/ml for IL-1β, IL-1ra, IFN-γ, IGF-1 and GFAP respectively.

### Statistical analysis

Normality of distribution was assessed with histogram plots and Kolgomorov–Smirnov test. Univariate analyses were performed using Student's *t*-tests, Mann–Whitney U-tests and chi-square tests. Multivariate analyses were performed using Kruskal–Wallis with pairwise post hoc comparisons. Correlations were with Spearman's Rho. Due to the low levels of detection of some cytokines, we conducted analyses using values below the manufacturer's LoD where sufficient cytokine was detected to do statistical analyses. These values were extrapolated from the standard curve. Due to the very low detectable levels of IL-1β, to avoid any undue bias from these extrapolated values, we performed two post hoc analyses. Firstly, we replaced all levels below the LoD with either a value of 0 (not detectable) or 1 (the limit of detection), and repeated the Mann–Whitney U test. Secondly, we created a dichotomous variable “IL-1β detected”, assigning a value of 1 or 0 according to whether or not the cytokine was measured above the manufacturer's LoD of 1 pg/ml, and comparing these rates of reliable detection between groups with or without delirium, using chi-squared tests. In order to adjust for potential confounders, a logistic regression analysis model was built for CSF IL-1β, using delirium at any stage as the dependent variable, using an Enter method. Co-variates were chosen if they were shown to be different between groups in the univariate analyses. The Statistical Package for the Social Sciences (SPSS) version 19.0 was used for data analysis. A value of p < 0.05 was considered significant.

## Results

Forty-three patients (32 females) were recruited from among a convenience sample within the study period, N = 24 in Edinburgh and N = 19 in Amsterdam. In both centres, this exploratory study was done in parallel with clinical work, with recruitment and sampling being carried out when the investigator was able to arrange this. Therefore the number of subjects was lower than expected because of logistical challenges. However, all patients meeting eligibility criteria and with samples were included in the analysis. Table 1 illustrates the baseline characteristics of the patient population. Mean age was 81.5 ± 7.5 years; cases and controls did not differ (Table 1). Delirium was present in 8/41 patients (20%) pre-operatively, 17/42 patients (40%) post-operatively and 19/43 patients (44%) at any stage. Nine of the 33 patients not delirious pre-operatively went on to develop delirium post-operatively (27%). A history of dementia (N = 8) was associated with a higher incidence of delirium (Table 1).

### CSF

Levels of cytokines in the CSF were generally low, with several below the manufacturer's recommended LoD, and some were mostly undetected. Table 2 illustrates the concentration of biomarkers detected in CSF. Delirium is classed according to whether it was present pre-operatively (prevalent), only developed post-operatively (incident) or never developed.

#### CSF IL-1β

IL-1β was detected in CSF at low concentrations: 20/44 (45%) of concentrations were above the manufacturer's recommended LoD of 1 pg/ml. Post hoc analyses reassigning results below the LoD of a value of either 0 or 1 did not alter the outcome of the results. Chi-squared analysis of whether or not IL-1β was detected in CSF revealed that if detected, it was more likely to come from a patient in the delirium group (Pearson chi-squared 3.79 p = 0.05). Using all values including those extrapolated from the standard curve, CSF IL-1β was higher in the group with delirium at any stage (median 1.02 pg/ml; IQR 0.66–1.74) compared to the no delirium group (median 0.66 pg/ml; IQR 0–1.02) (Mann Whitney U p = 0.02). Subgroup analysis revealed that CSF IL-1β was higher in patients with incident delirium (median 1.74 pg/ml; IQR 1.02–1.74) compared to prevalent delirium (0.84 pg/ml; 0.49–1.57) and those who never developed delirium (0.66 pg/ml; 0–1.02) (Kruskal–Wallis p = 0.03) (Table 2, Supplementary Fig. 1). Post hoc pairwise comparisons showed that this was due to the difference between incident delirium and never delirium groups (p = 0.03).

#### CSF IL-1ra

There was no difference in levels of CSF IL-1ra between those with (median 35.14 pg/ml; IQR 28.71–43.28) and without delirium at any stage (median 33.98 pg/ml; IQR 26.95–44.43) (Mann Whitney U p = 0.52). Subgroup analysis revealed that CSF IL-1ra was higher in patients with prevalent delirium (median 70.75 pg/ml; IQR 65.63–73.01) compared to those with incident delirium (31.06 pg/ml; 28.12–35.15) and those who never developed delirium (33.98 pg/ml (IQR 28.71–43.28) (Kruskal–Wallis p = 0.04)) (Table 2, Supplementary Fig. 2). Post hoc pairwise comparisons showed that this was due to the difference between incident and prevalent delirium groups (p = 0.04).

#### CSF GFAP, IFN-γ and IGF-1

No differences in GFAP levels were found in delirium at any stage (prevalent delirium median 0.81 ng/ml (IQR 0.33–1.31) vs. incident delirium 0.61 ng/ml (0.46–0.76) vs. never delirium 0.45 ng/ml (0.31–0.86), Kruskal–Wallis p = 0.58) (Table 2). IFN-γ and IGF-1 levels were all below the LoD in CSF and were not analysed further.

### Serum

Serum samples were available from 24 patients. Table 3 illustrates the concentrations of biomarkers detected in serum. Due to the smaller numbers in this exploratory substudy, delirium was classed as either present at any time or absent. IL-1β was found at concentrations above the LoD in 26% (6/23) of samples, and four of these samples were from the delirious group (Pearson's chi-square 2.58 p = 0.11). No differences were seen in serum levels of IL-1β, IL-1ra or IGF-1 with delirium at any stage (Table 3). IFN-γ and GFAP were below the LoD in serum, and were not analysed further.

### Correlation

Addressing the possibility that CSF IL-1β levels were a function of elevated IL-1β in the serum, we sought evidence for correlations between serum and CSF IL-1β, however there was no such correlation (Spearman's Rho 0.11, p = 0.62). An alternative hypothesis is that the combination of pre-existing dementia and subsequent hip fracture brought about CSF IL-1β production. Six of the eight (75%) dementia patients were CSF IL-1β positive and the other two had levels below 1 pg/ml but were detectable (0.32 and 0.66 pg/ml). Thirteen of the 34 patients without prior dementia were positive (38%) (Pearson chi-squared p = 0.06). Moreover, for those patients where both serum and CSF analyses were possible, we calculated CSF:serum ratios for IL-1β and found that this ratio was higher in the delirium group (delirium group N = 7, median ratio 1 (IQR 0.18–1.27), no delirium group N = 7, ratio 0 (0–0.48), MWU p = 0.02). There was no correlation between IL-1ra in CSF and serum (Spearman's Rho 0.24, p = 0.27) and no differences in IL-1ra CSF:serum ratio in groups with and without delirium (delirium group N = 8, median ratio 0.03 (IQR 0.02–0.07), no delirium group N = 15, ratio 0.06 (0.04–0.09), MWU p = 0.11). Serum IL-1ra and serum IL-1β levels were positively correlated (Spearman's Rho 0.50, p = 0.02). CSF IL-1ra correlated negatively with both serum IGF-1 (Spearman's Rho − 0.45, p = 0.03) and with CSF GFAP (Spearman's Rho − 0.48, p = 0.01).

### Logistic regression analysis

The only demographic variable shown to be different between groups with and without delirium at any stage was the presence of prior dementia (Table 1). A logistic regression model was built to adjust for the effect of the presence of prior dementia on the association between CSF IL-1β level and delirium status. The Wald statistic, odds ratios with 95% confidence intervals and P values for the logistic regression model are shown in Table 4, along with pseudo-R^2^ values. CSF IL-1β remained significant after adjusting for prior dementia status (Table 4). There was no evidence of collinearity, and assumptions for logistic regression were met.

## Discussion

These findings suggest an association of delirium with inflammation in the CNS. In particular, they implicate the IL-1β family in this process. IL-1β was increased in CSF in patients about to develop delirium, indicating that CNS IL-1β production may be an early event in delirium pathogenesis. IL-1ra levels were also increased in delirium, but only in patients who already had delirium pre-operatively, indicating that its production may be a later event in the delirium pathogenesis cascade.

A role for the IL-1β family in CNS inflammation underlying delirium pathogenesis is plausible. IL-1β is increased in CSF in CNS inflammatory disorders where there is manifest acute CNS damage, such as multiple sclerosis (MS) [25–27]. To put the levels of CSF IL-1β in the present study in a broader context, they were marginally higher than in acute stroke (0.80 pg/ml) [28], and similar to those in dementia; in Alzheimer's dementia levels range from 0.12 pg/ml [29] to 19.6 pg/ml [30], in vascular dementia 0.3 pg/ml [31] and in Dementia with Lewy Bodies 2.60 pg/ml [32]. Levels in age-matched controls range from 0.13 pg/ml to 23.3 pg/ml [30]. Levels in bacterial meningitis are several hundred folds higher, e.g. 410.1 pg/ml [33], 974 pg/ml [34]. Many studies reporting CSF IL-1β levels find them to be close to or below commercially-available assay LoD [30,32,35–37].

The source of CSF IL-1β could be peripheral, since IL-1β is capable of crossing from the periphery into the CNS via different routes [12–14], but given the lack of association between CSF and serum IL-1β levels in the current study, this is unlikely. Plausible sources of central IL-1β are macrophages of the choroid plexus and ventricular epithelium, brain vascular endothelial cells or microglia, the brain's resident macrophage population. All members of the IL-1 family (IL-1β, IL-1α and IL-1ra), their receptors and regulatory molecules are present in the brain, although expressed at very low levels in health [38]. It is now well established, in rodents, that systemic inflammation such as that caused by injury, surgery or infection, induces de novo CNS expression of IL-1β transcripts [2,3,39] and synthesis of IL-1β protein in circumventricular organs, the cerebro vasculature and in brain parenchymal microglial cells [17,40]. IL-1β is also produced quickly after various neurotoxic stimuli, predominantly by microglial cells, but occasionally by neurones, astrocytes, oligodendrocytes and endothelial cells [41,42]. Intrathecal production of IL-1β has been demonstrated in stroke patients [43]. That the central levels of IL-1β detected in this study were higher than the peripheral levels, with no correlation between the two, lends some weight to the suggestion that the source of CSF IL-1β is central, although the relatively small numbers and cross-sectional nature of the study make it difficult to infer source and causality. Furthermore, the increased CSF:serum IL-1β ratios in delirium versus no delirium offer tentative support for the microglial priming hypothesis that systemic inflammation produces exaggerated CNS inflammatory responses in those with prior CNS pathology and that this may have a role in delirium [2].

Interleukin-1β has pleiotropic actions in the CNS [44], and is strongly implicated in sickness behaviour [11,45,46]. Delirium has recently been suggested as a maladaptive form of this evolutionarily conserved, co-ordinated system of behaviour in vulnerable individuals [47]. Animal studies modelling delirium or acute cognitive dysfunction induced by systemic inflammation in aged rodents or those with prior neurodegeneration have consistently shown exaggerated CNS IL-1β responses to equivalent systemic stimulation [2–4,48]. Moreover, it has recently been shown that IL-1β is causative and synergises with cyclo-oxygenase 1-mediated prostaglandins to induce acute working memory deficits in an animal model relevant to delirium during dementia [5].

Physiological levels of IL-1β may be required for memory formation, with both deficiency and excess leading to decrements in hippocampal-dependent memory function in animal studies [42]. IL-1β is also thought to initiate a cascade of events leading to neuroinflammation, and in rodent models it exacerbates injury from other insults such as trauma or ischaemia [44]. IL-1β also has an effect on activation of the hypothalamic–pituitary–adrenal axis at all levels, leading to an increase in CNS cortisol levels [12,49]. Elevated CSF cortisol has recently been demonstrated in delirium [50]. Increased microglial expression of IL-1β has been reported in Alzheimer's disease [51]; this chronic expression is thought to contribute to plaque formation and precedes neurodegeneration [52]. IL-1β was found to be elevated in 75% of those with dementia in the current study, although importantly all of the patients had also suffered hip fracture, which would be predicted to drive IL-1β expression in the brain [39], particularly in those with prior CNS pathology [17]. The finding of elevated IL-1β in the CNS in delirium has biologically plausible implications in terms of the role of IL-1β in sickness behaviour, memory and neuroinflammation, and potential consequences in terms of neurotoxicity if this response is sustained.

We found elevated CSF IL-1β in those with incident delirium, that is, those who were about to develop delirium, often in the immediate post-operative period. This may be because significant generation or release of CNS IL-1β is an early phenomenon in delirium pathogenesis. Alternatively, post-operative delirium may have a different pathogenesis to pre-operative delirium, perhaps due to the more homogeneous insult of surgery and anaesthesia compared with potentially different precipitants in those who had perhaps fallen and fractured because they were already delirious, or developed delirium in association with pain, immobility, analgesia, fasting or dehydration in the pre-operative period.

The anti-inflammatory IL-1ra acts to inhibit IL-1β signalling through competitive binding [53]. It has been reported that IL-1ra production is delayed by 30 min compared to that of IL-1 β in response to ischaemia [54]. In this study, IL-1ra was higher only in patients with active delirium suggesting that this was after a rise in IL-1β, however in clinical terms, a 30 minute delay is unlikely to be relevant. It may be speculated that this production of IL-1ra is a neuroprotective response to the increased production of IL-1β; for example after traumatic brain injury, patients who mount a greater CSF IL-1ra response have a better neurological outcome [55], and IL-1ra is currently being trialled as a therapy in subarachnoid haemorrhage and severe traumatic brain injury [56,57]. It will be interesting to see if a rise in IL-1ra during a delirium episode correlates with a quicker or more complete recovery.

IL-1ra concentrations are required to be 50–100-fold higher than those of IL-1β to inhibit binding, as IL-1ra has a very short half-life of 4–6 h [58], and because very small concentrations of IL-1β are sufficient to activate its receptor [59]. In this study, IL-1ra concentrations were approximately 50-fold higher than IL-1β (Tables 2 and 3).

This study did not replicate previous findings of Adamis et al. of a decrease in serum IGF-1 [8]. In that study plasma measurements were taken at up to 4 time points during admission, while here the relatively small sample size and single blood collection may not have detected a decrease in IGF-1. CSF IGF-1 was not detected with the ELISA kit used, indicating that it is present at concentrations below the LoD of 26 pg/ml. Other authors have demonstrated levels of CSF IGF-1 in elderly control subjects of 4.30–4.40 pg/ml [60,61], and in elderly cohorts with Alzheimer's disease of 9.44 pg/ml [61], so it is possible that the ELISA we used was not sufficiently sensitive to detect IGF-1 in this setting.

IFN-γ was found to be below the LoD of 8 pg/ml in CSF and serum from all participants. Levels of CSF IFN-γ reported in the literature for healthy controls are 2.49 pg/ml [62] and 3.98 pg/ml [63]. IFN-γ was not detected in a cohort of patients with probable Alzheimer's disease, other dementias and cognitively normal elderly patients, with a LoD of 2 pg/ml [64]. CSF IFN-γ has been reported at levels of 53.53 pg/ml in herpes simplex encephalitis [35], 13.85 pg/ml in bacterial meningitis [63], and 4.26 pg/ml in MS patients within one week of acute relapse [63]. It is therefore likely that IFN-γ is present in low levels in CSF following hip fracture and in delirium. It is also possible that we did not detect a rise in IFN-γ in our cohort with hip fracture, since it is a sterile injury, as opposed to general medical cohorts in whom a rise in serum has been demonstrated previously, where the incidence of infection precipitating delirium may be higher [8].

Astrocytes, which express the IL-1β receptor, are involved in neuroinflammation, when they multiply and produce inflammatory intermediates [65]. Van Munster et al. have found increased GFAP staining in hippocampal sections from patients who died with delirium [66], but here we found no significant difference in CSF levels. CSF levels of GFAP were negatively correlated with CSF IL-1ra; if the presence of increased IL-1ra is a neuroprotective response, greater IL-1ra production may lead to less astrocyte activation. The levels we detected in our elderly cohort were similar to those found in younger patients with MS [67] and older patients with movement disorders [68], and lower than that seen in traumatic brain injury [69], and stroke [70]. GFAP is a structural protein of the cytoskeleton of fibrillary astrocytes, and its release into CSF is usually associated with acute CNS injury or astrogliosis [68,71].

Some limitations of the study should be addressed. Fifty-five per cent of IL-1β values were below the LoD of the assay. Although we tried to ensure that the use of extrapolated values did not substantially bias the findings, this may have been the case. One would also suspect a contribution of dementia to these findings, since premorbid dementia was strongly associated with the development of delirium peri-operatively and cytokine levels can be different in dementia. Importantly, the results of the logistic regression analysis suggest that CSF IL-1β remains associated with delirium after adjusting for the presence of prior dementia. In future studies the possibility of undiagnosed neurodegenerative disease should be addressed as robustly as possible with the use of informant history to assess premorbid cognition. An important strength of the methodology is the use of a planned urgent spinal anaesthetic to gain valuable CSF in a frail group with high rates of delirium, along with robust assessments for pre- and post-operative delirium. Since delirium fluctuates, it is always possible that we missed some mild or late cases, but we tried to minimise this by including casenote review and discussion with nursing staff.

In conclusion, this study supports the hypothesis that there are inflammatory mechanisms in delirium, and for the first time suggests a role for CNS IL-1β in the delirium pathogenesis cascade. A future larger study should confirm the increase in CSF IL-1β and IL-1ra. Analysis of longitudinal delirium data and multiple time points of sample collection will allow a thorough exploration of these and other cytokines putatively involved in delirium pathogenesis.

### Competing interests statement

All authors have completed the Unified Competing Interest form. The authors have no competing interests to report.

## Figures and Tables

**Table 1:** Baseline characteristics of patient group

	Delirium N = 19	No delirium N = 24	P value
Age	81.3 years (SD 6.0)	81.3 years (SD 8.6)	0.99^d^
Gender	Female N = 14 (74%)	Female N = 18 (75%)	0.92^e^
Hospital	Edinburgh (study 1) N = 3 Edinburgh (study 2) N = 6 Amsterdam N = 10	Edinburgh (study 1) N = 9 Edinburgh (study 2) N = 6 Amsterdam N = 9	0.29^e^
Type of fracturea,b	Femoral neck N = 9 Intertrochanteric N = 6 Periprosthetic N = 1	Femoral neck N = 8 Intertrochanteric N = 5 Periprosthetic N = 1	0.99^e^
Type of surgery	Hip replacement (bipolar or total) N = 10 Internal fixation N = 9	Hip replacement (bipolar or total) N = 10 Internal fixation N = 14	0.47^e^
Charlson co-morbidity index	5.00 (IQR 1.00–5.00)	3.50 (IQR 0.25–6.00)	0.79^f^
Prior dementia^c^	N = 7/17 (41.2%)	N = 1/24 (4.2%)	0.00^e^
Number of regular medications^a^	5.50 (IQR 3.25–10.5)	4.00 (IQR 1.00–6.00)	0.29^f^

Results expressed as median (interquartile range) or mean (standard deviation).

a Data not available for N = 12 Edinburgh study 1 participants.

b Data not available for N = 1 Amsterdam participant.

c Data not available for N = 2 participants.

d Student's *t* test.

e Pearson Chi-squared.

f Mann–Whitney U test.

**Table 2:** Concentrations of CSF markers in patients with delirium pre-operatively (prevalent), postoperatively (incident) and without delirium

CSF biomarker	Prevalent delirium	Incident delirium	Never delirium	P value
IL-1β (pg/ml)	0.84 (0.49–1.57) N = 8	1.74 (1.02–1.74) N = 9	0.66 (0.00–1.02) N = 24	0.03^a^
IL-1ra (pg/ml)	70.75 (65.63–73.01) N = 3	31.06 (28.12–35.15) N = 6	33.98 (28.71–43.28) N = 15	0.04^a^
GFAP (ng/ml)	0.81 (0.33–1.31) N = 8	0.61 (0.46–0.76) N = 9	0.45 (0.31–0.86) N = 24	0.58^a^

Results expressed as median (interquartile range).

a Kruskal–Wallis test.

**Table 3:** Concentrations of serum markers in patients with delirium at any stage or without delirium

Serum biomarker	Delirium at any stage	No delirium	P value
IL-1β (pg/ml)	0.66 (0.16–1.56) N = 9	0.00 (0.00–0.66) N = 14	0.08^a^ MWU = 35.0
IL-1ra (pg/ml)	1628.3 (SD 1086.7) N = 8	760.0 (SD 505.3) N = 15	0.06^b^ t = − 2.14
IGF-1 (ng/ml)	53.4 (SD 17.3) N = 9	68.3 (SD 36.9) N = 14	0.27^b^ t = 1.13

Results expressed as median (interquartile range) or mean (standard deviation).

a Mann–Whitney U test.

b Student's *t* test.

**Table 4:** Logistic regression model for CSF IL-1β

Co-variate	CSF IL-1β model			
	Wald	OR^a^	95% CI^b^ of OR^a^	P value
Prior dementia	5.64	0.06	(0.01–0.62)	0.02
CSF IL-1b	5.32	3.31	(1.20–9.15)	0.02
Constant	0.39	2.10		0.53

This table shows the results of the multivariate logistic regression analysis for CSF IL-1β, built to adjust for the potential confounding effect of prior dementia on delirium status. The dependent variable was delirium at any stage. Pseudo-R^2^ 0 (Cox and Snell), 0.31 (Nagelkerke) 0.42.

a OR: Odds ratio.

b CI: Confidence interval.

## Appendix A Supplementary data

Supplementary Fig 1. This dot graph shows levels of CSF IL-1β in groups with prevalent, incident or never delirium, indicating those participants with evidence of prior dementia. Supplementary Fig. 2. This dot graph shows levels of CSF IL-1ra in groups with prevalent, incident or never delirium, indicating those participants with evidence of prior dementia.
