# Supplementary figures and images for: Cerebrospinal fluid markers of neuroinflammation in delirium: A role for interleukin-1β in delirium after hip fracture
Source: J Psychosom Res. 2014 Sep;77(3):219–25. doi: 10.1016/j.jpsychores.2014.06.014 (PMC4274366; doi:10.1016/j.jpsychores.2014.06.014)

Figure 2

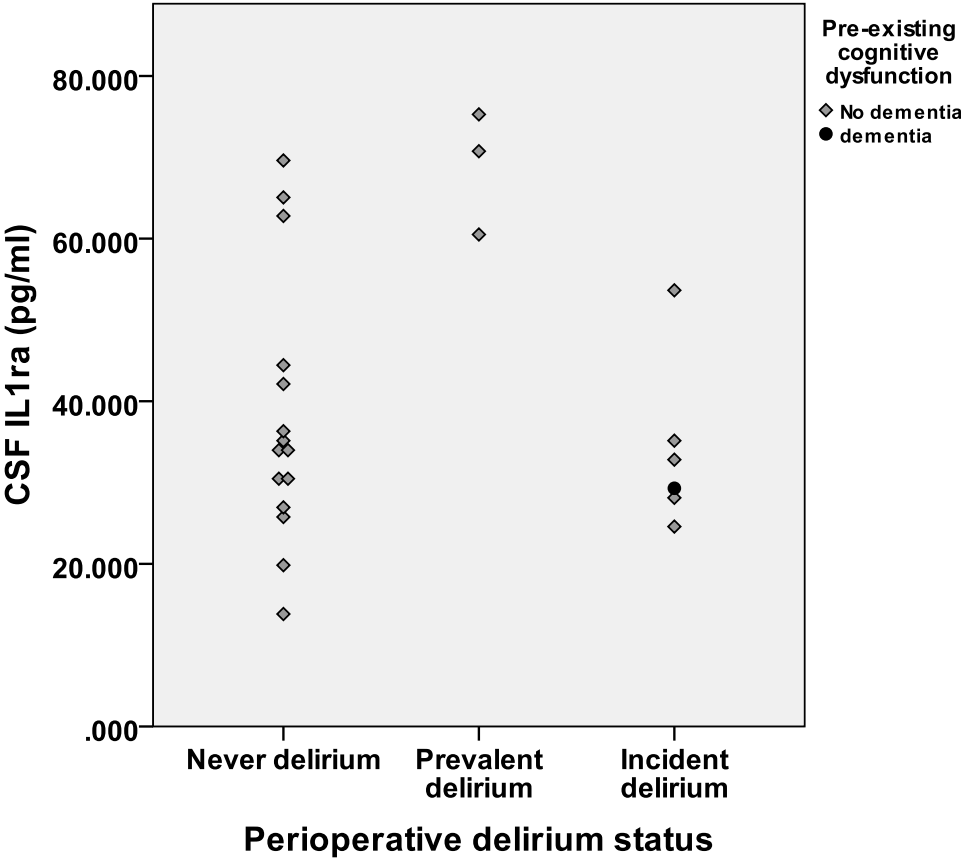

Supplement: Supplementary file 1 — Supplementary Fig 1. This dot graph shows levels of CSF IL-1β in groups with prevalent, incident or never delirium, indicating those participants with evidence of prior dementia. Supplementary Fig. 2. This dot graph shows levels of CSF IL-1ra in groups with prevalent, incident or never delirium, indicating those participants with evidence of prior dementia. [file mmc1.pdf]
